# Genetic Redirection of T Cells for the Treatment of Pancreatic Cancer

**DOI:** 10.3389/fonc.2019.00056

**Published:** 2019-02-12

**Authors:** Aesha I. Ali, Amanda J. Oliver, Tinaz Samiei, Jack D. Chan, Michael H. Kershaw, Clare Y. Slaney

**Affiliations:** ^1^Cancer Immunology Program, Peter MacCallum Cancer Centre, Melbourne, VIC, Australia; ^2^Sir Peter MacCallum Department of Oncology, University of Melbourne, Melbourne, VIC, Australia

**Keywords:** chimeric antigen receptor, pancreatic cancer, tumor microenvironment, pancreatic ductal adenocarcinoma, adoptive cell transfer, immunotherapy

## Abstract

Conventional treatments for pancreatic cancer are largely ineffective, and the prognosis for the vast majority of patients is poor. Clearly, new treatment options are desperately needed. Immunotherapy offers hope for the development of treatments for pancreatic cancer. A central requirement for the efficacy of this approach is the existence of cancer antigen-specific T cells, but these are often not present or difficult to isolate for most pancreatic tumors. Nevertheless, specific T cells can be generated using genetic modification to express chimeric antigen receptors (CAR), which can enable T cell responses against pancreatic tumor cells. CAR T cells can be produced *ex vivo* and expanded *in vitro* for infusion into patients. Remarkable responses have been documented using CAR T cells against several malignancies, including leukemias and lymphomas. Based on these successes, the extension of CAR T cell therapy for pancreatic cancer holds great promise. However, there are a number of challenges that limit the full potential of CAR T cell therapies for pancreatic cancer, including the highly immunosuppressive tumor microenvironment (TME). In this article, we will review the recent progress in using CAR T cells in pancreatic cancer preclinical and clinical settings, discuss hurdles for utilizing the full potential of CAR T cell therapy and propose research strategies and future perspectives. Research into the use of CAR T cell therapy in pancreatic cancer setting is rapidly gaining momentum and understanding strategies to overcome the current challenges in the pancreatic cancer setting will allow the development of effective CAR T cell therapies, either alone or in combination with other treatments to benefit pancreatic cancer patients.

## Introduction

Pancreatic cancer presents a major challenge in the clinic and is one of the most aggressive tumor types. Pancreatic cancer is the fourth most common cause of cancer death (1, 2) and it is on its way to be the second most common cause of cancer-related deaths by 2030 (3). Patients with pancreatic cancers have a median survival rate of 5 months after diagnosis and the overall 5-years survival rate is <5% (4).

The most common clinical therapeutic approaches against pancreatic cancer include surgical resection, radiotherapy, chemotherapy, and combination of these treatments (5). Surgical resection may lead to longer-term survival, but only a small number of the patients are considered resectable (6–8) because most patients that present to the clinic are with advanced or metastatic disease (9). In addition, the removal of part or the full pancreas is technically difficult, and even if the operation is successful, the 10-years survival rate is still <10% (10). Radiotherapy is usually not curative on its own and is used to alleviate symptoms. Although in the last two decades, chemotherapy and targeted therapy (such as Erlotinib targeting epidermal growth factor receptor, Sunitinib targeting multiple receptor tyrosine kinases, and Everolimus targeting mTOR kinase) have been used for patients with unresectable locally advanced or metastatic pancreatic cancer (11), these have only generated modest improvements in survival (12, 13). Therefore, the need to develop alternative effective therapies for pancreatic cancer is urgent.

Currently, immunotherapy has been offered as an important cancer treatment for a number of cancer types (14, 15) and recent preclinical and clinical evidence suggest that therapies utilizing the immunity could potentially be effective against this devastating disease (16). However, there has been limited success in the use of checkpoint blockade immunotherapies such as PD1/CTLA4 antibodies or vaccines in the treatment of pancreatic cancer (17).

Adoptive cellular therapies involving an infusion of effector immune cells into patients have generated remarkable responses in some cancers (18) and chimeric antigen receptor (CAR) T cell therapy represents a promising therapeutic modality for some difficult cancers including pancreatic cancers. This review aims to summarize the recent development of cellular therapies and clinical data in CAR T cell trials for pancreatic cancer. As pancreatic ductal adenocarcinoma (PDAC) is the most common malignancy of the pancreas and represents the vast majority of pancreatic cancer deaths, we will emphasize CAR T cell work on PDAC in this article.

## CAR T Cells

**Figure 1:**
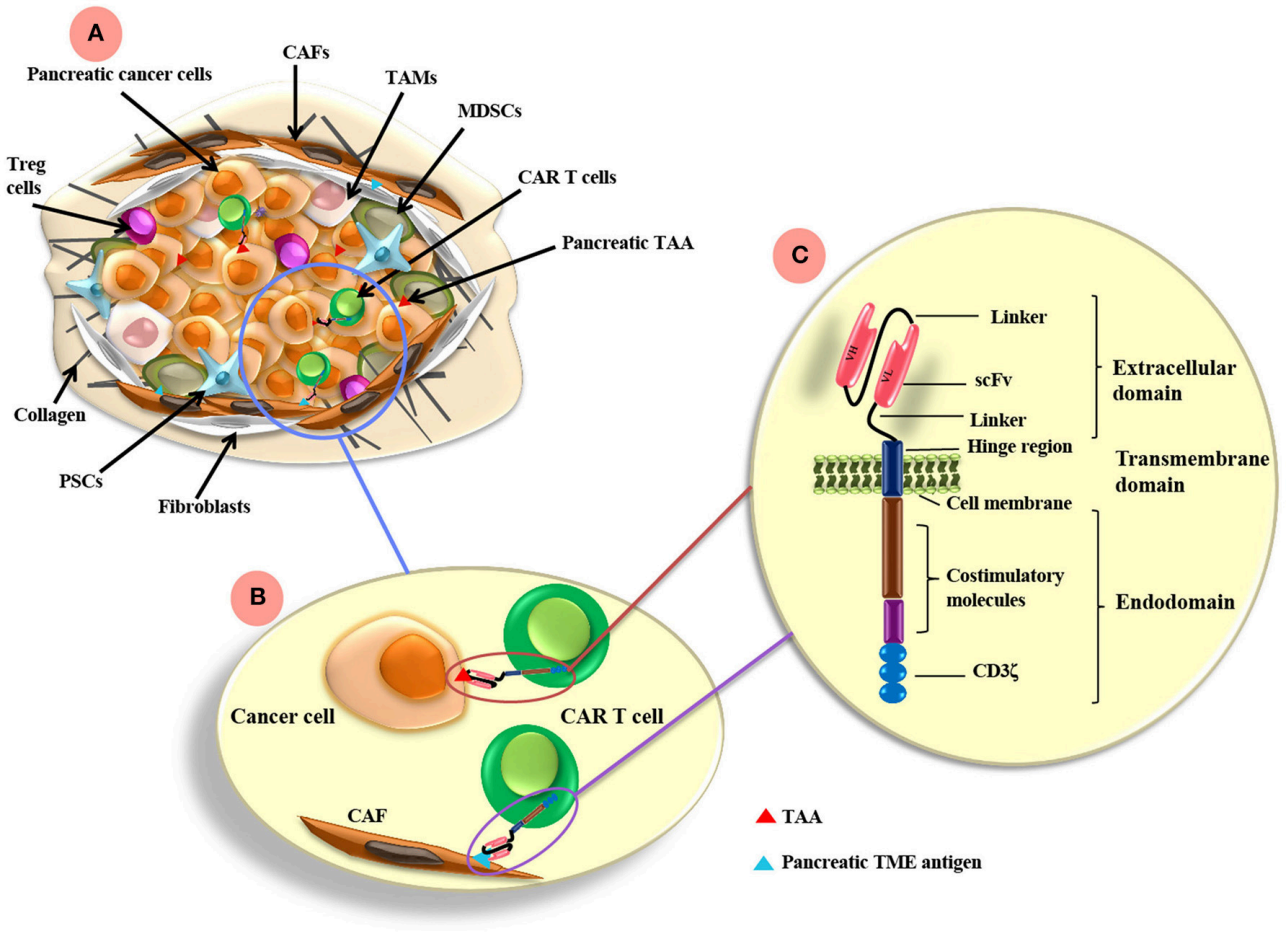
CAR T cell antigen-targeting strategies and pancreatic cancer TME. **(A)** The pancreatic TME consists of tumor cells as well as many immunosuppressive cells, such as CAFs, TAMs, MDSCs, PSCs, and Treg cells. **(B)** CAR T cells can be directed to the TAA expressed on pancreatic cancer cells and/or other antigens targeting the TME components, such as FAP on CAFs. **(C)** CARs are composed of extracellular, transmemebrane and endo-domains. The extracellular domain consists of an antibody variable heavy chain (VH) and a light chain (VL) domain, which are derived from an scFv from an antibody specific for a TAA. A flexible hinge region links the extracellular domain to a transmembrane and endodomain. The endodomain has cytoplasmic signaling regions derived from CD3ζ and costimulatory signaling domains. TAMs, tumor-associated macrophages; CAFs, cancer associated fibroblasts; MDSCs, myeloid-derived suppressor cells; Tregs, regulatory T cells; PSCs, pancreatic stellate cells; FAP, fibroblast activation protein; scFv, single chain variable fragment. TAA, tumor associated antigen; TME, tumor microenvironment.

CAR T cells present an exciting opportunity for cancer immunotherapy. As a form of adoptive immunotherapy, CAR T cells are generated from patient autologous T cells isolated from peripheral blood. Patient T cells are transduced *ex vivo* to express a CAR specific for a tumor antigen of choice and adoptively transferred into the patient to treat established cancers (19). CARs are composed of an antibody single-chain variable fragment (scFv) conjugated to intracellular signaling domains containing CD3-ζ chain and one or more co-stimulatory domains such as CD28 and CD137 (18, 20–22) (Figure 1). The CAR scFv confers the ability to T cells to directly recognize cancer antigens independent of MHC antigen presentation, and CAR specific recognition/binding to tumor antigen drives CAR T cell activation and tumor cell killing (23, 24). The first generation of CARs that was designed to contain CD3ζ or FcRγ signaling domains was limited by the lack of costimulatory signaling. The subsequent second generation of CARs has been designed to incorporate CD28 or CD137 cytoplasmic co-stimulatory domains. The third generation of CARs contains additional signaling domains (CD137, CD28, and/or OX40) (18, 20). The latter generations of CAR T cells are better equipped to overcome the immunosuppressive tumor microenvironment (TME), however, it remains unclear what combination of signaling domains is necessary for maximal anti-tumor response.

The use of CAR T cells for the treatment of B cell malignancies demonstrated significant responses in patients (25, 26). Given the success in clinical trials, the use of CD19-targeted CAR T cell therapies was approved by the FDA in 2017. Approved CAR T cell therapies include tisagenlecleucel (Kymriah) for the treatment of children and adolescents with refractory/relapsed B-cell acute lymphoblastic leukemia (B-ALL), and axicabtagene ciloleucel (Yescarta) for adult relapsed-refractory large B-cell lymphoma patients. However, despite the successes in hematological cancers, clinical trials targeting solid tumors have demonstrated only moderate efficacy. This is largely attributed to the immunosuppressive TME, limited activation and trafficking of CAR T cells to the tumor site, heterogeneous antigen expression/distribution in some solid tumors and availability of validated antibodies that could be utilized in the CAR constructs (27–29).

A range of approaches aimed at enhancing CAR T cell efficacy is currently undergoing investigation. A notable strategy that has demonstrated promising effects *in vivo* is the use of dual-specific T cells. Dual-specific T cells co-express a CAR against a tumor antigen and a TCR against a strong immunogen (30). Through vaccination, dual-specific T cells can engage the cognate immunogen of the chosen TCR presented by antigen presenting cells (APCs) on MHC molecules. A recent study using the “adoptive cell transfer incorporating vaccination” (ACTIV) therapy regimen for dual-specific T cell treatment has demonstrated durable responses in a range of solid tumors *in vivo* (31, 32). Use of the specialized “CARaMEL” dual-specific T cells, expressing a CAR against HER2 and TCR specific for the melanocyte protein gp100 (also known as pMEL), drove dramatic T cell expansion and tumor regression in a number of solid tumor models. Moreover, surviving mice that received ACTIV therapy developed potent immune memory responses against pre-existing tumor cells. These results provide encouraging evidence for the investigation and development of dual-specific T cells for the treatment of difficult cancers including pancreatic cancer.

## CAR T cell Therapies in Treating Pancreatic Cancer

In recent years, CAR T cell therapies have been tested in both preclinical and clinical settings for treating pancreatic cancers. However, a focus for the field remains the discovery and validation of pancreatic cancer-specific antigens.

### Mesothelin

Mesothelin (MSLN) is a glycoprotein present mainly in mesothelial cells and overexpressed in a variety of human cancers including malignant pleural mesothelioma, ovarian, lung and pancreatic cancers (33). MSLN has been reported to be expressed by >80% of PDACs and its expression correlated with poor prognosis (34). Although MSLN is believed to play a role in cell adhesion and positively regulates tumor invasion and growth, its biological function is unclear (34). Because MSLN is expressed only on non-crucial tissues, it is an attractive target for CAR T cell based immunotherapy.

Preclinical studies have demonstrated that CAR T cells against MSLNs could potentially be effective against PDAC. When MSLN-CAR T cells were adoptively transferred intratumorally or intravenously (i.v.) into NSG mice bearing pre-established subcutaneous patient-derived mesothelioma, the tumor burden was greatly reduced in size and some tumors were completely eradicated, demonstrating the potential of targeting this antigen (35). Due to the concerns for potential on-target/off-tumor toxicity, phase I clinical studies used mRNA-based methods to generate CAR T cells that express CARs transiently to limit the duration of toxicity. Given the short-term expression of CARs, multiple injections were required. In a study carried out by Beatty et al (36), a patient with metastatic PDAC was given eight doses of MSLN-CAR T cells by i.v. infusion and two doses via intratumoral injections. The CAR T cells were detected in the extravascular tumor compartments 3 days after the initial i.v. infusion. The patient demonstrated stable disease 3 weeks post MSLN-CAR T cell administration, without overt evidence of toxicity against normal tissues. An anti-tumor effect was observed based on the development of novel humoral immune responses post the cell infusion. In another phase I trial carried out by the same group, six metastatic PDAC patients were treated with mRNA-based MSLN-CAR T cells three times per week for three consecutive weeks. The treatment was well-tolerated and the disease was stabilized in two of the treated patients. Importantly, one patient had total metabolic active volume decreased by 69.2%, although there was no detected effect on the primary tumor (37). Given the promising results, trials of MSLN-CAR T cells engineered by traditional viral transduction methods have also been initiated (Table 1).

**Table 1:** Clinical trials involving CAR-based immunotherapy in pancreatic cancer from www.clinicaltrials.gov.

**Target antigen**	**Sponsor/collaborator**	**Cancer type**	**Status/estimated completion date**	**Number of patients**	**Trial ID**	**Notes**
MSLN	National Cancer Institute, USA	Pancreatic cancer Cervical cancer Ovarian cancer Mesothelioma Lung cancer	Recruiting/December, 2029	136 estimated	NCT01583686	Intravenous infusion of retroviral transduced MSLN-CAR T cells and low dose IL-2 with cyclophosphamide and fludarabine preconditioning.
	University of Pennsylvania, USA	Pancreatic cancer	Active, not recruiting/September, 2021	18 estimated	NCT03323944	1–3 × 10^7^/m^2^ (Cohort 1) or 1–3 × 10^8^/m^2^ (Cohort 2 and 3) lentiviral transduced MSLN-CAR T cells with/without cyclophosphamide preconditioning.
	Shanghai Gene Chem Co., Ltd./Shanghai Cancer Hospital, China	Metastatic pancreatic cancer	Unknown/July, 2018	20 estimated	NCT02959151	Intratumor injection or i.v. at one dose of 1.25~4 × 10^7^ CAR^+^ lentiviral transduced MSLN-CAR T cells/cm ^3^ tumor bulk.
	University of Pennsylvania, USA	Metastatic PDAC	Completed/March 2017	16 actual	NCT01897415	1–3 × 10^8^/m^2^ RNA transfected MSLN-CAR T cells i.v. injected three times weekly for up to 3 weeks. No cytokine release syndrome, neurologic symptoms or dose-limiting toxicities reported. One patient has a response in the live, but no activity in the primary tumor (37).
	University of Pennsylvania, USA	Metastatic PDAC Epithelial ovarian cancer Malignant epithelial pleuralmesothelioma	Completed/November, 2015	19 actual	NCT02159716	1–3 × 10^7^ and 1–3 × 10^8^/m^2^ lentiviral transduced MSLN-CAR T cells i.v. injected with or without cyclophosphamide preconditioning. Safe in patients with recurrent serious ovarian cancer. CAR T cells expanded in the blood of all patients. One patient had clearance of pleural effusion (104).
	Chinese PLA General Hospital, China	Malignant mesothelioma Pancreatic cancer Ovarian tumor Triple negative breast cancer Endometrial cancer Other MSLN^+^ tumors	Unknown/November 2018	20 estimated	NCT02580747	Retroviral transduced MSLN-CAR T cells.
	China Meitan General Hospital/Marino Biotechnology Co., Ltd. China	Mesothelin^+^ tumors	Recruiting/August 2019	20 estimated	NCT02930993	i.v. infusion with 5 × 10^4^-1 × 10^7^/kg MSLN-CAR T cells in a three-day split-dose regime. Cyclophosphamide preconditioning.
	University of Pennsylvania, USA	Pancreatic cancer Mesothelioma	Completed/September 2017	18 actual	NCT01355965	Three infusions of 1 × 10^8^-1 × 10^9^ mRNA MSLN-CAR T cells every other day for 2-cycle of three infusions. The pancreatic patient was given eight doses i.v. infusions and two intratumor injections. Anti-tumor effect observed and no overt toxicities (36).
	University of Pennsylvania/University of California, USA	Pancreatic cancer	Completed/November, 2017	4 actual	NCT02465983	i.v. infusion of 1–3 × 10^7^/m^2^ or 1 × 10^8^/m^2^ combined lentiviral transduced MSLN-CAR T cells and CD19-CAR T cells (to deplete B cells and impede the antibody response against MSLN CAR T cells) with cyclophosphamide preconditioning.
	First Affiliated Hospital of Wenzhou Medical University, China	Pancreatic cancer	Active, not recruiting/October, 2020	10 estimated	NCT03497819	Pancreatic arterial or i.v. infusion of lentiviral transduced MSLN-CAR T cells and CD19 CAR T cells with cyclophosphamide preconditioning.
	Shanghai GeneChem Co., Ltd. China	Pancreatic cancer	Unknown/September 2018	30 estimated	NCT02706782	Transcatheter arterial infusion of 1–10 × 10^6^ MSLN-CAR^+^ T cells/kg. Cyclophosphamide preconditioning.
MSLN, PSCA, CEA, HER2, MUC1, EGFRvIII and other targets	First Affiliated Hospital of Harbin Medical University/Shanghai Unicar-Therapy Bio-medicine Technology Co.,Ltd. China	Pancreatic cancer	Recruiting/June 2019	10 estimated	NCT03267173	A single dose of 10^7^ /kg CAR T cells administered i.v.
	Cancer Research UK, UK	Pancreatic cancer Breast cancer Colorectal cancer Gastric cancer Lung cancer Ovarian cancer Unspecified adult solid tumor	Terminated/April 2010	14 actual	NCT01212887	The pancreatic cancer patient received 10^9^-5 × 10^10^ retroviral transduced CEA CAR T cells i.v. and seven doses IL-2 with cyclophosphamide and fludarabine preconditioning. All patients had adverse events with grade ≤ 2 and lack of prolonged CAR T cell persistence led to the premature termination of the trial. No objective clinical response observed although serum CEA level reduced in the pancreatic cancer patient (47).
CEA	Roger Williams Medical Centre/University of Colorado, USA	Liver metastases Pancreatic cancer	Active, not recruiting/August, 2018	5 actual	NCT02850536	Three hepatic artery infusions of CEA-CAR T cells delivered by the Surefire Infusion System at 1-week intervals. Low dose IL-2 for 4 weeks.
	Southwest Hospital, China	Pancreatic cancer Lung cancer Colorectal cancer Gastric cancer Breast cancer	Recruiting/December, 2019	75 estimated	NCT02349724	CEA-CAR T cells were well-tolerated in high doses with some efficacy reported in the colorectal cancer cohort (105).
	Roger Williams Medical Center/Sirtex Medical, USA	Liver metastases	Active, not recruiting/January 2019	8 actual	NCT02416466	Three doses of CEA-CAR T cells and 1 dose of Selective Internal Radiation Therapy (SIRT) with Yttrium-90 at 2-weeks intervals combined with low dose IL-2 for 6 weeks.
PSCA	Bellicum Pharmaceutical, USA	PDAC Gastric adenocarcinoma Prostate adenocarcinoma	Recruiting/December, 2020	138 estimated	NCT02744287	i.v. infusion of retroviral transduced PSCA CAR T cells (BPX-601) with rimiducid (a homodimerizing molecule that enhances BPX-601 activation).
CD70	National Cancer Institute, USA	Pancreatic cancer Renal cell cancer Breast cancer Melanoma Ovarian cancer	Recruiting/January, 2028	113 estimated	NCT02830724	i.v. infusion of retroviral transduced CAR T cells. The CAR consists of the CD70 receptor CD27. Cyclophosphamide and fludarabine preconditioning with high dose IL-2.
MUC1	PersonGen BioTherapeutics (Suzhou) Co., Ltd./The First People's Hospital of Hefei; Hefei Binhu Hospital, China	Pancreatic carcinoma Hepatocellular carcinoma Non-small cell lung cancer Triple-negative invasive breast carcinoma	Unknown/October, 2018	20 estimated	NCT02587689	
	PersonGen BioTherapeutics (Suzhou) Co., Ltd./The First People's Hospital of Hefei/Hefei Binhu Hospital, China	Pancreatic carcinoma Hepatocellular carcinoma Non-small cell lung cancer Triple negative invasive breast carcinoma Malignant glioma of brain Colorectal carcinoma Gastric carcinoma	Unknown/July, 2018	10 estimated	NCT02839954	Anti-MUC1 CAR-NK cells.
HER2	Southwest Hospital, China	Pancreatic cancer Breast cancer Ovarian cancer Lung cancer Gastric cancer Colorectal cancer Glioma	Recruiting/September, 2019	60 estimated	NCT02713984	
EpCAM	First Affiliated Hospital of Chengdu Medical College, China	Pancreatic cancer Colon cancer Esophageal carcinoma Prostate cancer Gastric cancer Hepatic carcinoma	Recruiting/December, 2020	60 estimated	NCT03013712	1–10 × 10^6^ lentiviral transduced EpCAM-CAR^+^ T cells/kg. Vascular interventional or endoscopy infusion. Precondition before CAR T cell infusion.
Claudin 18.2	CARsgen Therapeutics, Ltd., China	Pancreatic carcinoma Adenocarcinoma of esophagogastric junction	Not recruiting/December, 2020	48 estimated	NCT03302403	i.v. infusion. Fludarabine and cyclophosphamide preconditioning.
	Changhai Hospital/CARsgen Therapeutics, Ltd., China	PDAC Advanced gastric adenocarcinoma	Recruiting/December 2021	24 estimated	NCT03159819	i.v. infusion of lentiviral transduced CAR T cells with lymphodepletion preconditioning.
CD133	Chinese PLA General Hospital, China	Pancreatic cancer Liver cancer Brain tumor Breast cancer Ovarian tumor Colorectal cancer Acute myeloid and lymphoid leukemias	Unknown/October 2018	20 estimated	NCT02541370	Retroviral transduced CD133-CAR T cells.

### Prostate Stem Cell Antigen

Prostate stem cell antigen (PSCA) is a glycosylphosphatidylinositol-anchored cell surface protein involved in intracellular signaling, although much of its function remains unclear. PSCA is expressed in the epithelial cells including that of prostate, kidney, skin, stomach, urinary bladder, esophagus and placenta, and also expressed in differentiating cells such as the ones of prostate and gastric epithelial cells. PSCA has also been detected in several cancer types including prostate, urinary bladder and pancreatic cancers (38). Aberrant overexpression of PSCA is detected in nearly 60% of the primary PDACs, while the gene expression is not detected in normal pancreatic duct (39). Therefore, PSCA has been proposed as a specific biomarker for PDAC patients and a promising target for CAR T cell therapies in treating PDAC. An advantage of targeting PSCA is that it is upregulated in pancreatic cancer cells from early stages of malignant transformation (40), including premalignant pancreatic intraepithelial neoplasias. PSCA may therefore serve as a useful target of immunotherapy that could eliminate malignant cells at all stages of PDAC.

Strategies using CARs against PSCA have been tested in preclinical settings. The 1st generation CAR T cells specifically killed PSCA^+^ pancreatic cancer cell lines without lysing PSCA^−^ target cell *in vitro* (8). In a more recent study, multiple CAR constructs were compared. Adoptive transfer of these human CAR T cells in this study demonstrated significant antitumor activity in a murine model of human pancreatic cancer. Interestingly, although the third-generation CAR containing CD28 and CD137 costimulatory domains induced greater persistence of CAR T cells *in vivo*, the second generation CAR that does not contain CD137 domain, induced a better antitumor effect, with 40% of mice demonstrating tumor eradication (40). The efficacy between second- and third- generation CARs have been compared by various studies and the discrepancies between these reports indicate that the optimal CAR design needs to be empirically determined for disease and antigen targeted (41, 42). Being encouraged by the preclinical success, a trial using CAR T cells against PSCA has been initiated and is currently recruiting patients (NCT02744287).

### Carcinoembryonic Antigen

Carcinoembryonic antigen (CEA) is a cell surface glycoprotein belonging to the immunoglobulin (Ig) superfamily and plays a role in cell adhesion. CEA is one of the “oncofetal” antigens, typically produced in the gastrointestinal tissue during fetal development (43, 44). Although CEA is expressed in various healthy epithelia of pulmonary and gastrointestinal tracts, its distribution is often limited to the luminal surface, thus difficult for CAR T cells to access (44). After neoplastic transformation, luminal epithelia cells lose the apical polarity of CEA expression and CEA becomes accessible to immune cells. Some CEA is released to the serum and soluble CEA in the circulation of patients is used as a marker for cancer progression. CEA is highly expressed on the surface of the majority of PDAC cells (45). Together with its restricted expression in normal tissues, CEA is an attractive target for CAR T cell treatment in PDAC. A few versions of anti-CEA CARs have been developed in the past few years, each targeting different CEA epitopes.

CEA-CAR T cells exhibited cytotoxicity against CEA expressing cancer cells *in vitro* and demonstrated anti-tumor effect *in vivo* in a clinically relevant orthotopic CEA^+^ murine model. The recipient CEA transgenic mice express CEA in their intestinal and pulmonary tracts. Ten days post intrapancreatic injections of Panc02-CEA^+^ cells, the recipient mice received CEA-CAR T cells. Interestingly, the injection of the CAR T cells eradicated tumors without any damage to normal tissues that are CEA^low^ (46).

However, a recent clinical trial using CEA-CAR T cells treating patients with advanced CEA^+^ cancers demonstrated acute respiratory toxicity, which resulted in the premature closure of the trial (47). The expression of CEA on lung epithelium was considered to result in the toxicity, which was associated with pre-conditioning. In a recent clinical trial using T cells modified to express an anti-CEA TCR for treating CEA^+^ metastatic colorectal cancer, severe autoimmune colitis and pneumonia was observed in all of the three patients and led to the halt of the trial (48). This was likely due to the ability of TCR-redirected T cells to engage CEA presented by MHC.

### Mucin 1

Mucins are high-molecular-weight glycoproteins with the presence of a heavily *O*-glycosylated tandem repeat region that is rich in proline, threonine and serine residues. The large gel-forming mucins are an extracellular secretion of goblet cells and their functions include lubrication of the epithelial surfaces and protection from physical and chemical insult. The epithelial membrane-tethered mucins are distinct from the conventional secreted mucins and are transmembrane molecules expressed by most glandular and ductal epithelial cells. It is widely accepted that the transmembrane mucin 1 (MUC1) is overexpressed in multiple epithelial adenocarcinomas, such as that of breast, colon, and pancreatic cancers. Importantly, under normal conditions, MUC1 is heavily glycosylated and expressed on the apical surface of epithelial cells, but in tumor cells MUC1 is aberrantly glycosylated. This modification of the MUC1 antigen reveals epitopes associated with the core protein, which is usually masked by oligosaccharides. Overexpression of aberrantly glycosylated MUC1 is associated with multiple metastatic cancers. In particular, MUC1 is aberrantly expressed in 60% of pancreatic cancers and is correlated with poor prognosis, enhanced metastasis, and chemoresistance (49, 50).

Strategies using CAR T cells targeting aberrantly expressed MUC1 have generated exciting results in preclinical studies. Posey et al. (51) generated a CAR that recognizes the aberrant glycoform Tn (GalNAca1-O-Ser/Thr) antigen on MUC1. These CAR T cells are able to recognize multiple types of tumors *in vitro* and exhibited superior tumor rejection and prolonged survival against disseminated pancreatic cancers in a xenograft model. This elegant study highlighted the potential for protein modifications as a target. As one of the most characteristic features of cancer cells is altered glycosylation, changes in glycosylation may expose a range of different cancer-associated epitopes and can serve as a target for CAR T cells. Given the promising outcomes from preclinical studies, multiple early phase trials have been planned and are active for using MUC1-CAR T cells in treating PDAC (Table 1).

### CD47

CD47 is a transmembrane protein known to mediate a “do not eat me” signal. Structurally, CD47 contains an extracellular N-terminal hydrophilic Ig superfamily domain and an intracellular hydrophobic domain. Signal regulatory protein alpha (SIRPα) has been identified as the receptor to CD47. The binding of tumor-expressing CD47 to SIRPα on immune cells leads to the activation of the downstream signaling pathway in immune cells and inhibits the immune phagocyte-dependent clearance of tumors. CD47 has been identified in several types of hematological and solid cancers including pancreatic cancer (52, 53). In addition, CD47 is expressed on high levels on cancer stem cells (CSCs), but not on normal cells in the pancreas (52). Therefore, targeting CD47 has been a subject of intense interest in recent years.

CD47-specific CAR T cells were recently developed by ProMab Biotechnologies. These CAR T cells demonstrated high cytotoxicity against a few types of cancer cells including pancreatic cancer cells. Importantly, intratumoral injection of these CAR T cells significantly decreased pancreatic xenograft tumor growth. The same research group also developed humanized CD47-CAR T cells that contain humanized CD47 scFv. These humanized CD47-CAR T cells demonstrated specific killing of CD47^+^ cancer cells *in vitro* and it will be interesting to assess their efficacy in clinical trials (54).

### Tyrosine Kinase Growth Factor Receptors

The tyrosine kinase growth factor receptors are transmembrane proteins that play a key role in medicating intracellular signal transduction cascade for cell proliferation and differentiation. Up-regulation of some of the receptors are mechanisms of cancer development and progression, and the overexpression of a number of these receptors have been identified in many types of cancers (55). Therefore, CARs designed to target tyrosine kinase growth factor receptors have been developed and clinical trials are under way to test the safety and efficacy of many of these targets.

Human epidermal growth factor receptor 2 (HER2, also known as ERBB2) is a transmembrane glycoprotein belonging to the epidermal growth factor receptor (EGFR) family. The binding of HER2 to its ligand induces heterodimerization of the receptors, which mediates the activation of intracellular tyrosine kinase signaling cascades and leads to cell proliferation and differentiation. Overexpression of HER2 induces dimerization of HER2 and initiates signal transduction without ligand binding. Overexpression of HER2 has been reported in multiple cancer types making it an attractive target for CAR T cell treatment (20). However, an early HER2-CAR T cell trial, utilizing a third generation CAR, reported a patient death post the CAR T cell treatment (56). This patient was diagnosed with colon cancer metastatic to the lung and liver. The patient was preconditioned using cyclophosphamide (CY) and flurodarabine. Following intravenous infusion of 10^10^ HER2-CAR cells in 30 min, the patient experienced acute respiratory distress and subsequent death. At autopsy, multiple organs including the lung showed signs of ischemia and injury, and her serum samples post infusion showed significant increased levels of multiple cytokines, including IFN-γ, IL-6, and TNF-α. This report raised safety concerns on targeting HER2, but a more recent study using lower numbers of HER2-CAR T cells, and a second generation CAR in treating 19 patients with HER2^+^ sarcoma, demonstrated safety (57).

HER2 expression is observed in 20–60% of pancreatic cancer cases and therefore, it is a potential target for CAR T cell treatment (58–61). Recently, a Phase I clinical trial used HER2-CAR T cells to treat two pancreatic cancer patients. Although the trial demonstrated safety, only moderate responses were achieved (62). In contrast, results from preclinical studies have been promising. A recent study used HER2-CAR T cells to treat mice bearing xenografts derived from stage IV PDAC patients and achieved complete remission in both local and disseminated disease settings. In addition, in this study, the authors used an antibody-based switchable CAR system. These switchable CAR T cells bind to a specific peptide that is genetically engrafted onto a tumor-binding Fab molecule. The switch acts as a bridge between the tumor cells and CAR T cells and has a short half-life and thus, limits potential immunogenicity. The comparison between the switchable and conventional CAR T cells demonstrated that the anti-tumor efficacy of these switchable CAR T system was not compromised (63). The discrepancy in efficacy results from clinical and preclinical studies may be due to the different levels of HER2 expression in patients and xenografts, and the immunosuppressive TME in the patients.

Other putative growth factor receptors that could be targeted against pancreatic cancer using CARs include insulin-like growth factor receptor-1 (IGF1R), EGFR, vascular endothelial growth factor receptor (VEGFR), fibroblast growth factor receptor (FGFR) and platelet-derived growth factors (PDGFRs) that are expressed at elevated levels in pancreatic cancers and contribute the cancer's malignant phenotype (55). IGF1R is expressed in a variety of cancers and blocking IGF1R expression enhances apoptosis and suppresses metastasis in pancreatic cancer cells (64). IGF1R-CAR cells have demonstrated efficacy in murine models of sarcoma xenograft (65) and could be potentially useful in the treatment of PDAC. EGFR is a surface glycoprotein that belongs to the EGFR family of tyrosine kinase receptors. EGFR is aberrantly activated in a number of epithelial tumors and its overexpression has been detected in up to 90% of pancreatic tumors (66, 67). The value of EGFR-CAR T cells in treating solid cancers has been demonstrated in both preclinical (68) and clinical settings (69) in a few different cancer types but its potential in treating PDAC is yet to be tested.

### CD24

CD24 is a mucin-like protein. It was originally discovered as the ligand for P-selectin and involved in signal transduction mediated by the members of the protein tyrosine kinase family. CD24 expression is observed in over 70% of PDAC tumors and in putative PDAC cancer stem cells (CSC). CSC can also express CD44 and epithelial specific antigen (ESA) or CD133. Given the prevalence of early-disseminated metastases, CSCs are believed to play a key role in the pancreatic cancer development and progression. Among these PDAC CSC protein markers, CD24 has the lowest expression in normal tissue and thus is proposed as a suitable target antigen for immunotherapy (70, 71). A study carried out by Maliar et al. examined the therapeutic efficacy of both HER2-CAR and CD24-CAR in treating PDACs in murine models. In mice bearing subcutaneous human PDAC cell line Capan-1 (positive for both HER2 and CD24), intratumor injection of the CAR T cells targeting either HER2 or CD24 greatly inhibited tumor growth, in some cases, eradicated tumors. In mice bearing orthotopic Capan-1 tumors, i.v. injections of these two different CAR T cells also demonstrated anti-tumor effects to both primary and metastatic tumors at the liver and lymph nodes. Interestingly, the cells that were dissociated from patient pancreatic cancers for xenograft in this study, displayed a heterogeneous expression pattern of antigens, including both HER2 and CD24. The adoptive transfer of HER2-CAR or CD24-CAR to mice bearing these xenografts significantly arrested the tumor growth, but to a different degree, dependent on the CAR-targeted antigen specificity. The results from this study highlight the antigen heterogeneity nature of human pancreatic tumors (72). It remains to be seen if the administration of CAR T cells targeting both antigens will enhance efficacy. This study highlights potential therapeutic limitations using CARs targeting a single tumor antigen in treating complex cancers that are heterogeneous in antigen expression patterns and distributions.

### Fibroblast Activation Protein

Fibroblast activation protein (FAP) is a type II integral membrane serine protease. Healthy adult tissues have no detectable FAP expression. However, under certain biological circumstances, such as remodeling, wound healing, and embryogenesis, FAP expression has been observed. FAP is also present in a large proportion of tumor stromal fibroblasts in the majority of epithelial carcinomas including pancreatic cancers (73) and its expression correlates with poor prognosis in pancreatic cancer patients (74). The carcinoma-associated fibroblasts (CAFs) are a central player in tumorigenesis and metastasis and the key characteristics of CAFs is the expression of FAP (75). Due to its high expression in CAFs, FAP has been tested as a CAR target.

Tran et al. investigated the use of FAP-CAR T cells targeting tumor stromal fibroblasts in a number of mouse tumor models and human pancreatic cancer xenografts. Despite *in vitro* activity observed, the injection of the FAP-CAR T cells only elicited limited *in vivo* anti-tumor effect. Unexpected side effects such as cachexia and lethal bone toxicities were observed. The off-target effect was due to the expression of FAP on murine bone marrow stromal cells (BMSCs). In this study, human BMSCs were also identified in expressing FAP and could be recognized by FAP-CAR T cells (76). The finding that FAP is expressed by BMSCs raised safety concerns for therapies targeting FAP.

Interestingly, in separate studies carried out by Wang et al. (77) and Kakarla et al. (78) using FAP-CAR T cells, no toxicity was observed. The reason may be due to the different scFvs used. The scFv used by Wang et al. targets a different FAP epitope and only eliminate FAP^hi^ cells, while sparing FAP^low^ cells including BMSCs.

## Clinical Trials Using CAR T Cells Against Pancreatic Cancer

A substantial number of clinical trials involving the use of CAR technology have recently been undertaken on pancreatic cancer patients in an attempt to investigate the potential safety, effectiveness and feasibility of the approach. A variety of tumor associated antigens (TAA) expressed on pancreatic cancer cells have been targeted in various clinical trials to redirect CAR T cells against pancreatic cancer including MSLN, CEA, PSCA, MUC1, and HER2 (Table 1).

A range of CAR formats are used in these clinical studies, although chiefly second-generation formats, with either CD28 or CD137 cytoplasmic domains. Transduction methods also vary, including electroporation with CAR-encoding RNA, but viral vectors, either retroviral or lentiviral, are the main method of CAR gene delivery (Table 1). Typically, dose escalation is used in these trials, which aim to determine safety, with doses ranging from 1 × 10^7^ to 3 × 10^8^ per m^2^. Higher doses of RNA transfected T cells are used, since they present less of a risk due to their limited duration of CAR expression. The majority of studies involve a single dose of CAR T cells; some trials use multiple doses (Table 1).

There is substantial variation in the use of preconditioning, although some level of lymphodepletion is proposed for most studies. The combination of CY and fludarabine is often preferred to induce a deep level of lymphodepletion to enhance engraftment of transferred CAR T cells. While most of the studies detailed in Table 1 involve the transfer of CAR T cells alone, some propose to use additional drugs to enhance CAR T cell activity. Thus, IL-2 is proposed for some studies to provide T cells with a supporting growth factor.

To enhance the effectiveness and the safety of CAR T cell therapy, some clinical trials use reagents that activate CAR T cells. For example, in trial NCT02744287 targeting PSCA, a dimerizer agent, Rimiducid (AP1903) is used. Rimiducid is administered with PSCA-specific CAR T cells that contain an inducible MyD88/CD40 (iMC) costimulatory domain. In another trial (NCT02416466), radiation is delivered using Yttrium-90 microspheres to maximize the tumoricidal effects of CAR T cells and minimize the effects on healthy liver parenchyma in patients with liver metastases.

The majority of clinical trials of CAR T cells in pancreatic cancer are in early stages of recruitment, but some have been completed and initial reports are available. CAR T cells are generally well-tolerated in pancreatic cancer patients, although some toxicity was reported when targeting CEA (47). Despite the early nature of most trials, there are some reports of CAR T cell efficacy. The CARsgen trial reported their early results at the 2018 CAR-TCR Summit in Boston that using an anti-Claudin-18.2 CAR treating pancreatic and gastric cancer patients resulted in some objective responses without overt toxicities (79, 80). However, there are no descriptions of significant responses in most trials to date, some unique challenges presented by pancreatic cancer may have to be overcome to maximize responses.

## Tumor Microenvironment as a Unique Challenge for CAR T Cells in Pancreatic Cancer

A major obstacle for immunotherapies, in particular CAR T cell therapies, in solid tumors is the immunosuppressive tumor microenvironment (TME) (75). The TME impacts on the efficacy of CAR T cells both by limiting their infiltration and suppressing their function within the tumor (29, 81).

A unique feature of the pancreatic TME is the desmoplastic stromal reaction, which promotes tumor growth and provides a physical barrier for therapeutic drugs and T cell infiltration. In fact, PDAC is one of the most stroma-rich cancers and in some cases, the stromal components precede pancreatic cancer cells. In normal pancreas, the pancreatic stellate cells (PSCs) are a rare population and function to store retinoids in a form of lipid droplets in the cytosol. During pancreatic cancer progression, the pancreatic stellate cells (PSCs) become activated by tumor-secreted cytokines, lose retinoid droplets and transform into a myofibroblast phenotype. The activated PSCs secrete extracellular matrix (ECM) proteins and deposit collagens to form a dense fibrotic cancer stroma (82). In addition, activated PSCs secrete cytokines (such as IL-6 and IL-11) and chemokines (such as CXCL12, CCL5, CCL2, and CCL17) that recruit immunosuppressive leukocyte subsets (82–85). Indeed, as discussed above, CAR T cells that target the highly expressed CAF protein, FAP, are considered in pancreatic cancer due to the significant role these cells play in tumorigenesis (77, 86, 87) and in addition, FAP is positive in PDAC-derived ECMs but negative in normal PSCs (88). The particularly harsh TME is a major barrier to CAR T cell efficacy in pancreatic cancer and thus additional modulators will be required for durable responses (Figure 1).

Approaches that enable CAR T cells to sustain and function in the TMEs have been investigated in a large number of preclinical and clinical studies in a range of malignancies, and the results have revealed various potential strategies against pancreatic cancers. For example, depleting immunosuppressive cell subsets such as Tregs, MDSCs and TAMs has demonstrated enhanced efficacy of CAR T cell therapies (29). These suppressive immune cell types are enriched in pancreatic cancer and are associated with increased tumor growth and poorer prognosis (89–91). Various reagents that have shown to eliminate these suppressive immune cells or modulate their functions have been tested in murine models in recent years, such as IL-2 toxin and anti-CD25 (for Treg depletion), CSF-1R inhibitor and CCR2 toxin (for MDSCs/TAMs) (92–95). A few anti-TAM drugs such as antibodies against CCL2, CSF1R that inhibit the recruitment and survival of TAMs and MDSCs are currently under clinical investigation (96). Strategies of using CARs against these suppressive cells are emerging. For examples, inserting CSF1R-CARs into NK and T cells for killing TAMs have demonstrated promising outcomes *in vitro* (97). In our laboratory, using ACTIV therapy that involved injecting dual-specific CAR T cells and a vaccine, TAMs decreased significantly post the treatment, coinciding with tumor regression (32). In humans, the engraftment of T cells is enhanced with myeloablative preconditioning regimes and thus specific depletion methods could enhance CAR T cell efficacy in pancreatic cancer patients (98, 99).

Another strategy for enhancing CAR T cell efficacy in solid tumors is blocking the inhibitory signals received by the T cells from immunosuppressive populations in the TME. Checkpoint inhibitors, against molecules such as PD-1, CTLA4, TIM-3, and LAG-3 have shown promise as single agents in a number of cancer types (100) but unfortunately, none of these treatment as a single therapy has generated significant clinical benefit in pancreatic cancers. CAR T cells can express high amounts of these checkpoint molecules, which can lead to apoptosis and hypo-function. Results from preclinical studies clearly demonstrated that CAR T cell treatments could benefit from the addition of checkpoint inhibitors (101, 102). Some early clinical data also support the use of a combination of the checkpoint inhibitors with CAR T cell therapy in treating difficult cancers. A study carried out by Chong et al. reported that in a diffuse large B cell lymphoma patient refractory to CART19, after PD-1 blockade, the patient had an expansion of the CAR T cells and clinically significant antitumor response (103).

In addition, a number of strategies have been tested to enhance CAR cells to sustain in a suppressive cytokine milieu. Methods such as introducing the CD137 signaling domain within the CAR intracellular domain to increase mitochondrial biogenesis (104), expressing regulatory subunit I anchoring disruptor (RIAD) peptide to CAR T cells to disrupt protein kinase A (PKA) activation (105), and constitutively expressing CD40L by CAR T cells (106) have all demonstrated potential in enhancing CAR T cell treatment efficacy in solid cancers. A recent elegant study combined MSLN-CAR T cells with an oncolytic adenovirus expressing TNF-α and IL-2 to treat human PDAC xenograft models and a sygeneic mouse tumor model. This strategy significantly enhanced CAR T cell anti-tumor efficacy. The anti-tumor effect was linked to increased tumor-infiltrating lymphocytes and altered TME including altered polarization of macrophages and maturation of dendritic cells (107).

Given the limited options for efficacious pancreatic cancer treatment and the success of CAR T cells in hematological malignancies, in which TME differs in its degree of immunosuppression, overcoming this obstacle in pancreatic cancer will be an important consideration for future research.

## Conclusions and Future Perspectives

Pancreatic cancer is the most lethal cancer and new therapies are urgently needed. CAR T cell therapy represents a revolutionary treatment for cancers and has generated remarkable responses in hematological malignancies. The extension of CAR T cell therapy into pancreatic cancer recently started and this field is moving forward rapidly. Due to its unique immunosuppressive TME and antigen complexity and heterogeneity (108), pancreatic cancer presents one of the most difficult cancers for immunotherapies. Understanding the pancreatic cancer TME and how this TME affects CAR T cell efficacy is key in designing effective CAR T cell treatments. In addition, besides expanding the CAR-antigen landscape, targeting multiple antigens simultaneously (109) and using strategies targeting neoantigens (16) could provide significant opportunities for treating pancreatic cancer.

## Author Contributions

AA, MK, and CS: conception and design; AA, AO, TS, JC, MK, and CS: write, review, and revision of the manuscript; MK and CS: supervision.

### Conflict of Interest Statement

The authors declare that the research was conducted in the absence of any commercial or financial relationships that could be construed as a potential conflict of interest.
